# Current Advances in Wearable Devices and Their Sensors in Patients With Depression

**DOI:** 10.3389/fpsyt.2021.672347

**Published:** 2021-06-17

**Authors:** Seunggyu Lee, Hyewon Kim, Mi Jin Park, Hong Jin Jeon

**Affiliations:** ^1^School of Medicine, Sungkyunkwan University, Seoul, South Korea; ^2^Department of Psychiatry, Hanyang University Medical Center, Seoul, South Korea; ^3^Department of Psychiatry, Depression Center, Samsung Medical Center, Seoul, South Korea; ^4^Department of Health Sciences and Technology, Department of Medical Device Management and Research, Department of Clinical Research Design and Evaluation, Samsung Advanced Institute for Health Sciences and Technology, Sungkyunkwan University, Seoul, South Korea

**Keywords:** wearable devices, sensors, major depression, biomarkers in psychiatry, mood monitoring

## Abstract

In this study, a literature survey was conducted of research into the development and use of wearable devices and sensors in patients with depression. We collected 18 studies that had investigated wearable devices for assessment, monitoring, or prediction of depression. In this report, we examine the sensors of the various types of wearable devices (e.g., actigraphy units, wristbands, fitness trackers, and smartwatches) and parameters measured through sensors in people with depression. In addition, we discuss future trends, referring to research in other areas employing wearable devices, and suggest the challenges of using wearable devices in the field of depression. Real-time objective monitoring of symptoms and novel approaches for diagnosis and treatment using wearable devices will lead to changes in management of patients with depression. During the process, it is necessary to overcome several issues, including limited types of collected data, reliability, user adherence, and privacy concerns.

## Introduction

The rapid development of wearable devices has led to their active use in research on depression. Sensors in wearable devices can collect physiological data related to mental health. These devices enable monitoring and assessment of patients in real time and in an unobtrusive way. In addition, the data collected through wearable devices can be monitored by the patient, and health care providers can receive and use the patient-generated health data to personalize healthcare. It is expected that the big data obtained by wearable technologies will facilitate the delivery of personalized, interactive, noncontact healthcare in a cost-effective manner.

Studies have shown that some types of data obtained from various wearable devices concern depression. Vallance et al. (1) reported that greater physical activity as measured by an accelerometer was correlated with lower rates of depression. Other research found that skin conductance as measured by wearable patches can be a sensitive biomarker for depression (2–4). A meta-analysis reported that heart rate variability (HRV), which is defined as spontaneous fluctuations in heart rate mainly reflecting the activity of the autonomic system, is reduced in patients with depression, even without concomitant cardiovascular disease (5, 6). HRV often is measured by photoplethysmography (PPG) to determine changes in microvascular perfusion by illuminating the skin with light and measuring the transmitted or reflected amount (7).

Although the market of wearable devices is growing rapidly, the use of wearable technology for diagnosis and treatment of depression remains limited. Therefore, the purpose of this study was to provide a current overview of the developments and utility of wearable devices in research on depression. Here, we collected original studies examining wearable devices for diagnosis and treatment of depression and highlight the developments and trends in this field.

## Methods and Results

To identify studies on the use of wearable devices in depression research, a literature search was performed in PubMed and the Web of Science databases, focusing on articles published prior to December 31, 2020. The specific search string was as follows: (wearable^*^ OR actigraph^*^ OR actigraphy OR actiwatch OR actimetry OR smartwatch OR wrist-worn OR “fitness tracker” OR “inertial sensor” OR “digital outcome measure”) AND (depress^*^ OR MDD OR “major depressive disorder” OR bipolar OR unipolar OR “affective disorder” OR “mood disorder”). This search string was applied only to article titles.

**Figure 1:**
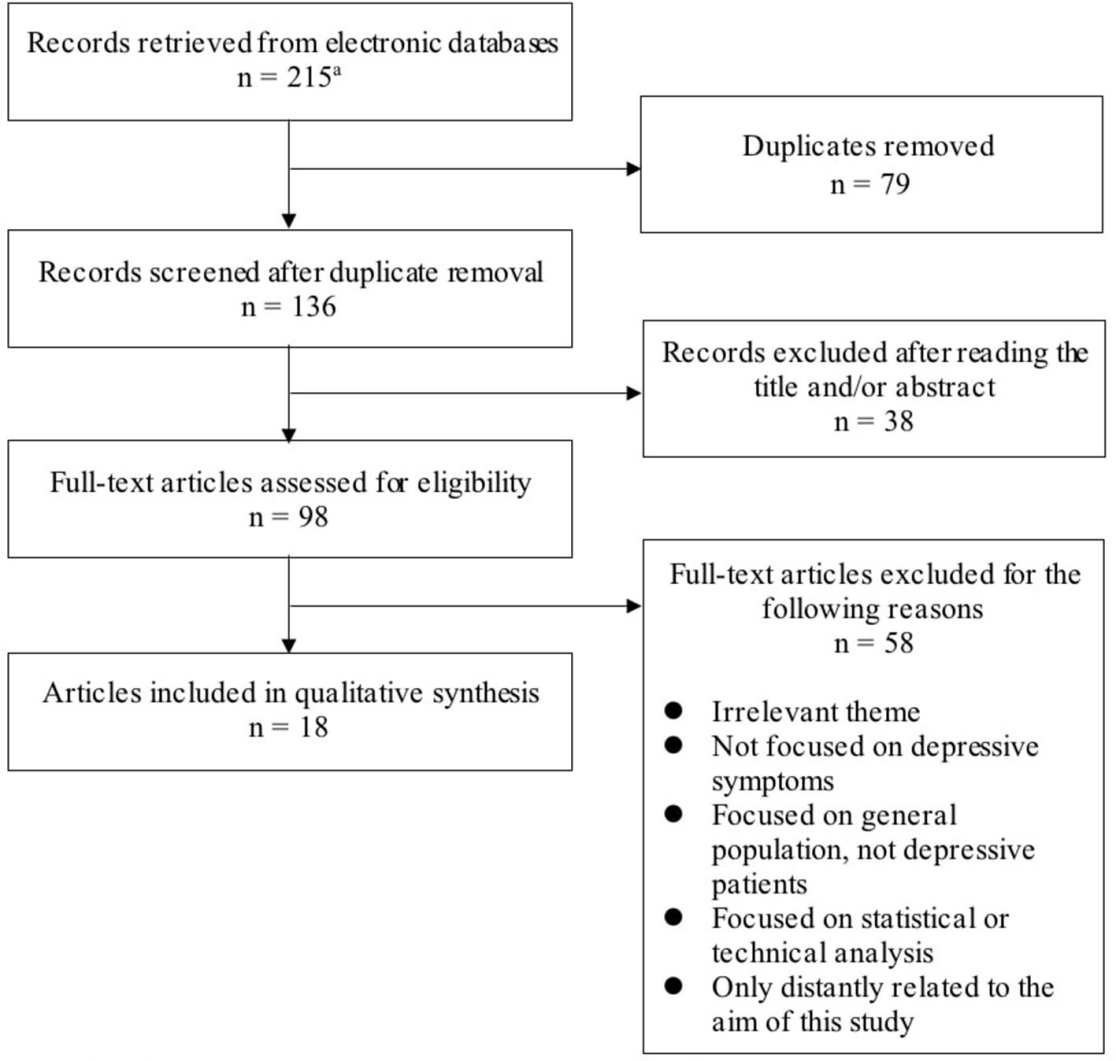
Flow diagram of the literature search. ^a^PubMed/Medline: *n* = 89; Web of Science: *n* = 126.

For this study, we excluded papers not written in English and the following types of manuscripts: conference papers, meeting abstracts, research notes, brief/short reports, letters, corrections, protocols, reviews, systematic reviews, meta-analyses, and editorial materials. We also excluded papers with an irrelevant theme (e.g., wearable bipolar batteries) or that were only distantly related to the aim of this study. In addition, we excluded papers that were not focused on depressive symptoms or focused on the general population or statistical and technical analysis. The search process is presented in Figure 1. We collected a total of 18 studies that made use of wearable devices to assess or monitor depressive symptoms or to predict major depressive disorder (MDD) (Table 1).

**Table 1:** Clinical trials with wearable devices in research on depression.

**References**	**Subjects**	**Devices**	**Methods**	**Main points**
Raoux et al. (8)	Inpatients with MDD (*n* = 26).	Wrist actigraph	24-h motor activity pattern monitoring at Days 0 and 28.	Activity level was increased after pharmaceutical treatments.
Winkler et al. (9)	Outpatients with seasonal affective disorder (*n* = 17).	Wrist actigraph	4 weeks of activity monitoring with BLT in the morning.	BLT normalized disturbed activity patterns and restored circadian rhythms in seasonal affective disorder patients.
Chung and Tso (10)	Patients during an acute episode of MDD (*n* = 91).	Wrist actigraph	Actigraphic data collected twice over a 3-month period.	Sleep data measured by actigraphy may predict pain symptoms in MDD.
Razavi et al. (11)	Medicated inpatients with MDD (*n* = 76).	Wrist actigraph	24-h actigraphic monitoring.	Motor-related single item “activities” of HAMD were associated with motor activity parameters, while the total score was not.
McCall and McCall (12)	Patients with a current major depressive episode and chronic insomnia (*n* = 54).	Wrist actigraph	Overnight study with concurrent actigraphic and PSG monitoring.	There were moderate positive correlations between actigraphy and PSG for all variables.
Rothschild-Fuentes et al. (13)	MDD outpatients aged 60 years or more (*n* = 10).	Wrist actigraph	Actigraphic parameters recorded before mirtazapine treatment and at day 60 of the treatment.	Sleep fragmentation index was significantly decreased after mirtazapine treatment, while other sleep parameters were not significantly changed.
Winkler et al. (14)	Inpatients with treatment-resistant depression.	Wrist actigraph	Activity level measured before and after ECT.	Remitters had increases of light activity, total activity, and circadian amplitude.
Hoogerhoud et al. (15)	Severely depressed patients (*n* = 12).	Wrist actigraph	5-day actigraph monitoring during ECT course.	Actigraphy-assessed sleep in the short-term was not affected by ECT.
Krane-Gartiser et al. (16)	MDD inpatients with and without motor retardation (*n* = 25 and 27).	Wrist actigraph	24-h actigraphy recordings.	Reduced mean activity level, higher intraindividual variability, and lower complexity were shown in patients with motor retardation compared with patients without motor retardation.
Nishida et al. (17)	Patients with medication-resistant MDD.	Waist actigraph	Monitoring over the course of rTMS treatments.	Sleep variables did not show significant changes, but *post-hoc* tests indicated a significant increase in mean steps per day.
O'Brien et al. (18)	Adults with late-life depression and aged 60 years or more (*n* = 29).	A novel wrist-worn device with three accelerometers	Monitoring over 7 days.	Subjects with late-life depression showed significantly reduced physical activity and slower fine motor movements.
Cook et al. (19)	Patients with unipolar MDD (*n* = 21).	Fitbit Flex™	An overnight study with concurrent actigraphic and PSG monitoring.	The Fitbit Flex™ is not adequate to be substituted for PSG when evaluating sleep in MDD.
Cormack et al. (20)	Patients with mild-to-moderate MDD (*n* = 30).	Apple watch	Cognition and depressed mood assessment by new Cognition Kit app every day over 6 weeks.	Daily mood and cognitive assessments correlated moderately with validated tests.
Rojo-Wissar et al. (21)	Adults with MDD (*n* = 34).	Wrist actigraph	Self-reported parental bonding instrument and wrist actigraphy (for 1 week) were evaluated.	Sleep characteristics in adulthood were associated with maternal bonding but were independent of depression status.
Tazawa et al. (22)	Depressed patients (*n* = 45).	Silmee W20 wristband	Machine learning models developed using data collected by the device over seven days.	Skin temperature and sleep parameters were the most significant features for prediction.
Powell et al. (23)	Patients with severe unipolar or bipolar depression (*n* = 12)	PKG	PKG used to assess motor symptoms in depression.	PKG measures were significantly correlated with clinically assessed melancholia.
Peis et al. (24)	Depressed patients (*n* = 23).	Wrist actigraph	Regression model was developed to predict clinical course and hospital discharge of depressed patients.	Increased motor activity and early patterns of actigraphic measures allowed for accurate prediction of hospital discharge date.
Pedrelli et al. (25)	Patients with MDD (*n* = 31).	Empatica E4 wristband	Assessment by smartphone, wristband sensors, in-person clinical interviews, HDRS for 8 weeks.	The predicted score of the developed model and clinician-rated HDRS showed moderate-to-high correlation; skin conductance, HRV, and activity were important features of the model.

## Discussion

### Overview

Among the collected studies on wearable devices for patients with depression, two-thirds used actigraph units, while the rest used other devices such as a novel wearable device with three accelerometers (18); medically used wearable devices, such as a Parkinson's KinetiGraph (PKG) (23) and the E4 wristband (Empatica, Boston, MA, USA) (25); and commercial wearable devices not originally intended for medical use, such as the Fitbit Flex™ (Fitbit, Inc., San Francisco, CA, USA) (19), Apple watch (Apple Inc., Cupertino, CA, USA) (20), and Silmee™ W20 wristband (TDK Corporation, Tokyo, Japan) (22). Among the reviewed studies, no wearable devices were used for treatment. In all studies, specific physiological, activity/sleep, or subjective parameters of individuals were collected through wearable devices, and the relationship between these parameters and depression was investigated. Except for one study that employed a waist actigraph unit (17), all included studies used wrist-worn devices to assess their study populations. Because wearing accessories on the wrist is nondistracting and familiar to most people, wrist-worn wearable devices have been used actively for research.

The use of actigraphy for monitoring depressive symptoms has been investigated since the 1990s and involves using wristwatch-like devices to collect activity or sleep data generated by movements (26). An actigraph unit is typically equipped with linear or a three-axis accelerometer to detect movements. Actigraph units are produced by various companies, with most studies (9, 11, 14, 15) using actigraph units manufactured by Cambridge Neurotechnology Ltd (Cambridgeshire, England). However, new devices and algorithms are being developed constantly, and there is no consensus regarding which device or algorithm is most appropriate for assessing patients with depression (26).

Some relevant medical devices include PKG and the Empatica E4 wristband. The PKG is a wristwatch-like device with a three-axis accelerometer originally designed to assess motor symptoms such as bradykinesia and tremor in patients with Parkinson's disease (27). However, Powell et al. (23) recently used it to assess motor symptoms in patients with depression. On the other hand, the Empatica E4 is a wrist-worn device that contains a PPG sensor capable of detecting heart rate, an electrodermal activity (EDA) sensor, an optical thermometer for measuring peripheral skin temperature, and the three-axis accelerometer for estimating motion and sleep characteristics. This device was used by Pedrelli et al. (25) to monitor changes in depressive symptom severity of patients.

Some consumer devices not originally indicated for medical use are the Fitbit Flex™, Apple watch, and Silmee™ W20 wristband. The Fitbit Flex™ is a commercially available fitness tracker equipped with a three-axis accelerometer that measures motion patterns of users. The utility of the Fitbit Flex™ in evaluating sleep patterns in depressive patients was compared against polysomnography (PSG) and actigraphy (19). On the other hand, the Apple watch is a so-called “smartwatch” featuring various apps as well as capabilities for phone calls and text messaging. It contains a small touchscreen, which was used by Cormack et al. (20) to offer tasks like cognitive and mood assessments to study participants. Finally, the Silmee™ W20 wristband was used previously (22) for unintended medical use and is equipped with a three-axis acceleration sensor, pulse sensor, ultraviolet sensor, and temperature sensor.

### Measured Parameters

Various parameters related to depression have been measured by each wearable device and can be classified as activity/sleep, physiological, and subjective parameters.

Activity characteristics, sleep patterns, and circadian rhythm can be estimated by accelerometers in wearable devices. Previous studies have consistently revealed the association between physical activity and MDD. People who do not regularly engage in physical activity are more likely to show depressive symptoms (28), and depression can be a significant risk factor for a sedentary lifestyle (29). Most studies employing wearable devices have reported results consistent with these statements. When inpatients with MDD were treated with antidepressants, their activity level as measured by actigraphy was significantly increased upon discharge (8). Elsewhere, Winkler et al. (9) observed that activity was increased even by means of electroconvulsive therapy (ECT). Finally, Peis et al. (24) reported that increased motor activity and early patterns of actigraphic measures allowed for accurate prediction of hospital discharge date using a Hierarchical Generalized Linear Regression Model.

In a previous study, MDD patients with motor retardation had decreased level of activity compared with those without motor retardation (16). Meanwhile, Powell et al. (23) showed the potential for PKG to be used to evaluate movement patterns such as bradykinesia in patients with MDD.

Insomnia is a risk factor for depression, and sleep-related parameters can be used for prediction of depression (30). In addition, depressive patients tend to report more unsatisfactory sleep quality than healthy controls (31). However, this finding was not supported by most studies using actigraphy for sleep assessment of depressive patients, even though McCall and McCall (12) showed that actigraphic measurement of sleep is closely approximated those of PSG. Specifically, with mirtazapine medication for MDD patients, no significant improvement was found for actigraphic sleep measures (13). Also, both Winkler et al. (14) and Hoogerhoud et al. (15) reported that ECT did not affect actigraphy-assessed sleep. Rojo-Wissar et al. (21) suggested that maternal bonding characteristics were associated with sleep characteristics in adulthood but were not dependent on depression status. However, unlike other studies, Chung and Tso (10) showed that insomnia symptoms measured by actigraphy can be used to predict pain symptoms in MDD patients by Pearson correlation analysis, so further research is needed.

Several studies have shown that circadian rhythm–related parameters are correlated with depressive symptoms. Winkler et al. (9) reported that circadian rhythms in patients with seasonal affective disorder can be restored with bright light therapy (BLT). In another study, remitters had increases in circadian amplitude (the gap between the peak and the mean of a wave), but no significant changes were observed in circadian acrophase (the time at which the peak of a rhythm occurs) (14).

Skin temperature (ST), skin conductance (SC), and HRV as measured by an EDA sensor, optical thermometer, and PPG sensor, respectively, are physiological parameters collected by wearable devices. Tazawa et al. (22) reported that ST can be an indicator of depression using a machine learning model with parameters detected from the Silmee™ W20 wristband. This corresponds with the report that depressive patients have a higher body temperature than that of healthy controls (32).

The study by Pedrelli et al. (25) corresponds with previous research suggesting that SC is significantly correlated with depression (33), and that HRV reflects autonomic dysregulation affected by mental health status (34). They have proposed a machine learning model that predicts clinical scores of MDD from various data collected through a smartphone and wristband. According to them, the 10 most predictive features for MDD include parameters related to SC and HRV. However, further research is necessary since machine learning models usually remain black boxes and do not clearly conclude to what degree and why the underlying parameters are related to the prediction (35).

### Future Trends

The advantage of incorporating wearable devices into research in patients with depression is that wearable devices enable continuous and objective monitoring of patients. With this technology, real-time changes between patient hospital visits can be tracked objectively and treatment effects can be monitored more accurately. Assessments of MDD often are conducted by mood questionnaires such as the Hamilton Depressing Rate Scale (HDRS). However, these types of mood assessments are easily disturbed by various biases, such as recall bias (36). This can lead to inaccurate results depending upon the degree of inconsistency among patients or clinicians (37). In addition, errors that can occur during manual data entry by physicians or researchers can be prevented when using wearable devices.

Wearable devices also enable patients to monitor their symptoms. For example, a patient can check their current level of stress or depression using wearable devices or a smartphone connected via Bluetooth. In a study using Psymate (PsyMate BV, Maastricht, the Netherlands), a “personal digital assistant”–based system for mood assessment, personalized feedback interventions appeared to help patients improve their depressive symptoms and prevent maladaptive behaviors that can worsen their moods, suggesting that the provision of such feedback through wearable devices will have similar effects (38). It has shown that, if patients are notified about their mood frequently, it may help them to manage their own depression.

Wearable devices can potentially be used to develop novel diagnostic methods or treatments. Sensors can measure various parameters other than those introduced above; for example, speech pattern and voice analyses through sensors can be used to assess depression severity and treatment response (39). In addition, using sensors like inertial sensors, bending sensors, and electromyography signals, it is possible to capture human motion through wearable devices (40). Considering studies showing that depressive patients can exhibit gait variability (41) and motor abnormalities (42), future research is expected to use novel wearable devices to investigate how movement changes with depression.

Recently, wearable devices that can treat depression at home have been developed. One study (43) showed that daily morning light therapy for 60 min using home light-therapy glasses can trigger an improvement in depressive symptoms. If this technology can be applied to normal glasses with light sensors tracking the daily light exposure of patients, daily use can help to monitor and treat depression.

Wearable devices can be used as a part of personalized exercise-treatment programs for MDD (44). In addition, wearable devices can increase patient physical activity and medication adherence according to an ongoing trial enrolling patients with heart failure and diabetes mellitus (45); based on step count data from wearable devices in this study, individualized feedback is provided to patients. We can expect a study in the future that attempts this type of intervention for patients with depression.

An aripiprazole tablet with an ingestible sensor (Abilify MyCite; Otsuka America Pharmaceutical, Inc., Rockville, MD, USA) was approved recently by the United States Food and Drug Administration. The sensor on the pill sends a signal to a cutaneous wearable so that clinicians can track the medication adherence of patients. This model is expected to be used in depression studies considering that aripiprazole can be administrated as an adjunctive treatment for depression.

### Challenges

There are challenges in the application of wearable devices in the field of depression. First, it is difficult to detect various symptoms of depression using wearable devices. In particular, evaluation of subjective mood symptoms is difficult; for example, wearable devices mostly detect physiological data and have limitations in evaluating subjective symptoms. However, a recent study using the Apple watch showed the possibility of high-frequency cognitive and mood assessments using wearable devices (20). Second, physiological data that can be measured by wearable devices are lacking. For example, although a change in appetite is a common symptom in patients with depression, it is difficult to measure it with wearable devices. Vu et al. (46) previously tried to estimate eating behaviors by detecting wrist movements or the sound of eating, but the accuracy was low.

There are various issues with adherence and compliance of patients. Wearable devices have variations in the degree of convenience of use, and there is a difference in user comfort when wearing the devices. The effectiveness of mobile health interventions varies greatly according to design of the intervention (47), and acceptance of wearable devices can vary depending on user age (48). Therefore, further research is required about how to increase adherence and compliance rates so that users can continuously wear wearable devices for 24 h.

Unreliability and inaccuracy are problems in use of wearable devices. Various wearable devices for fitness and wellness are available on the market. However, since these devices are heterogeneous, they are not simple to use for those wishing to monitor clinical symptoms. The reliability of the sensor system and data-processing algorithm of wearable devices also makes it difficult to introduce these devices into the medical field. Furthermore, data can be disrupted by various kinds of noises generated by the surrounding environment and the physical condition of the person wearing the device. Although some studies have been carried out to validate the accuracy of wrist actigraphy in comparison with PSG (49, 50), accelerometers on the wrist are not effective in detecting sleep patterns not involving limb motion, so other instruments like a pressure sensor sheet or chest-worn sensor are needed to obtain a higher degree of accuracy comparable to that of PSG (51, 52).

Another issue involves feature extraction with regard to unreliability issues. According to a layered hierarchical framework presented by Mohr et al. (53), raw sensor data (e.g., location, movement) are transformed into low-level features (e.g., activity, total sleep time). The low-level features are combined and constitute high-level behavioral markers (e.g., psychomotor activity, sleep disruption). The clinical state (e.g., depression) is inferred through a combination of high-level behavioral markers. Although it is a logically valid framework, whether the data, such as the movements measured with an accelerometer, can accurately represent psychomotor agitation or retardation is unclear. For example, sleep is estimated based on patient motion and pulse not by measuring their brainwaves (54).

There also is a problem of privacy and ethics. Since data obtained through wearable devices are stored on an external server, there is a possibility that this data can be leaked. Because of this problem, legal regulations for use in the medical field will be essential.

## Conclusion

There are rapid ongoing developments of wearable devices for clinical use. Depressive symptoms can be estimated by many parameters collected objectively in real-time by wearable devices. The possibility of diagnosis and prediction remotely using these devices has been supported in this review. Future trends are expected with the emergence of new wearable devices that will bring novel diagnostic and therapeutic approaches like motion capture, speech analysis, and portable light therapy. This suggests the potential for fundamental changes in diagnosis and treatment of depression in the future by developments of wearable devices. These developments can lead to early and accurate diagnosis of depression, the capability to provide more personalized treatment to patients with depression, and to develop preventive measures for groups at risk of depression. Wearable devices will have a critical role in medicine with the advent of personalized telemedicine.

## Author Contributions

HK, MP, and HJ contributed to conception and design of the study. HK and MP performed the bibliographical search. SL wrote the first draft of the manuscript. SL, HK, MP, and HJ wrote sections of the manuscript. All authors contributed to the article and approved the submitted version.

## Conflict of Interest

The authors declare that the research was conducted in the absence of any commercial or financial relationships that could be construed as a potential conflict of interest.
